# Scaling DEPP phylogenetic placement to ultra-large reference trees: a tree-aware ensemble approach

**DOI:** 10.1093/bioinformatics/btae361

**Published:** 2024-06-13

**Authors:** Yueyu Jiang, Daniel McDonald, Daniela Perry, Rob Knight, Siavash Mirarab

**Affiliations:** Electrical and Computer Engineering Department, University of California San Diego, 9500 Gilman Dr, La Jolla, CA, 92093, United States; Pediatrics Department, University of California San Diego, 9500 Gilman Dr, La Jolla, CA, 92093, United States; Pediatrics Department, University of California San Diego, 9500 Gilman Dr, La Jolla, CA, 92093, United States; Pediatrics Department, University of California San Diego, 9500 Gilman Dr, La Jolla, CA, 92093, United States; Center for Microbiome Innovation, Jacobs School of Engineering, University of California San Diego, 9500 Gilman Dr, La Jolla, CA, 92093, United States; Electrical and Computer Engineering Department, University of California San Diego, 9500 Gilman Dr, La Jolla, CA, 92093, United States; Center for Microbiome Innovation, Jacobs School of Engineering, University of California San Diego, 9500 Gilman Dr, La Jolla, CA, 92093, United States

## Abstract

**Motivation:**

Phylogenetic placement of a query sequence on a backbone tree is increasingly used across biomedical sciences to identify the content of a sample from its DNA content. The accuracy of such analyses depends on the density of the backbone tree, making it crucial that placement methods scale to very large trees. Moreover, a new paradigm has been recently proposed to place sequences on the species tree using single-gene data. The goal is to better characterize the samples and to enable combined analyses of marker-gene (e.g., 16S rRNA gene amplicon) and genome-wide data. The recent method DEPP enables performing such analyses using metric learning. However, metric learning is hampered by a need to compute and save a quadratically growing matrix of pairwise distances during training. Thus, the training phase of DEPP does not scale to more than roughly 10 000 backbone species, a problem that we faced when trying to use our recently released Greengenes2 (GG2) reference tree containing 331 270 species.

**Results:**

This paper explores divide-and-conquer for training ensembles of DEPP models, culminating in a method called C-DEPP. While divide-and-conquer has been extensively used in phylogenetics, applying divide-and-conquer to data-hungry machine-learning methods needs nuance. C-DEPP uses carefully crafted techniques to enable quasi-linear scaling while maintaining accuracy. C-DEPP enables placing 20 million 16S fragments on the GG2 reference tree in 41 h of computation.

**Availability and implementation:**

The dataset and C-DEPP software are freely available at https://github.com/yueyujiang/dataset_cdepp/.

## 1 Introduction

Phylogenetic placement of a query sequence on a backbone tree is increasingly used (Matsen *et al.* 2010, Mirarab *et al.* 2012, Zheng *et al.* 2018, Barbera *et al.* 2019, Linard *et al.* 2019, Mai and Mirarab 2022, Balaban *et al.* 2020, Turakhia *et al.* 2021, Wedell *et al.* 2023). Placement can identify taxonomic groups making up a biological sample, a problem that is consequential in many downstream applications. Placement is used extensively in microbiome analyses (Janssen *et al.* 2018) and tracking epidemics (Turakhia *et al.* 2021). What placement offers, in lieu of the *de novo* reconstruction, is scalability: since placement processes queries independently, it scales linearly with the number of queries and enables analyzing millions of queries. This focus on scalability, however, should not come at the expense of accuracy.

A main lesson learned in analyses using existing tools, one that should not be surprising, is that the accuracy of the placements and downstream analyses both depend on the density of the backbone tree (e.g., Nasko *et al.* 2018, McDonald *et al.* 2023). For example, Balaban *et al.* (2020, 2022) documented that subsampling a larger tree to create smaller backbone trees reduced accuracy for all methods tested. Most methods have reduced accuracy when the closest matches in the reference database differ substantially from the query. This observation has spurred the development of many reference sets (some using genome-wide data) that include tens to hundreds of thousands of taxa (e.g., Quast *et al.* 2012, Shi *et al.* 2019, Parks *et al.* 2018, Zhu *et al.* 2019, Asnicar *et al.* 2020, McDonald *et al.* 2023). These large databases include a fraction of available prokaryotic genomes and a tiny fraction of an estimated 10^12^ microbial species (Locey and Lennon 2016).

While placement methods are naturally scalable with more queries, they do not always scale to large backbone trees. This lack of scalability has motivated the development of booster methods such as pplacer-XR (Wedell *et al.* 2021) and SCAMMP (Wedell *et al.* 2023) that scale existing methods. These methods rely on a divide-and-conquer strategy that breaks the backbone into smaller subsets. Some of these methods, such as APPLES-II (Balaban *et al.* 2022) and SCAMMP, have been successfully run with reference trees with tens of thousands of leaves with reasonable running times.

Traditional placement methods use some model of sequence evolution to place a query on a tree that is assumed to have generated the sequences. A new paradigm some of us recently proposed (Jiang *et al.* 2022a) is to use the species tree as the backbone while sequence data come from a single or a handful of genes. While this so-called discordant placement is conceptually less appealing than the traditional approach, it is useful in practice. The goal of sample identification is to find species identities, not genes; discordant placement makes that conflict explicit. Also, by allowing updates of a species tree using single genes, it provides a path for combining two types of data historically analyzed separately: genome-wide (shotgun) metagenomic data and 16S amplicon-based data. If we can place 16S data on the species tree, we can jointly analyze 16S and genome-wide data. Jiang *et al.* (2022a) demonstrated that this goal is achievable with decent accuracy. In particular, they proposed a metric learning framework for phylogenetic placement using deep neural networks (NN). The proposed method, DEPP, trains a model based on a backbone tree and a multiple sequence alignment (MSA); it uses the model to place new queries, which need to be aligned to the backbone MSA. The trained model is an embedder that maps sequences onto the $R^{d}$ space using an NN such that Euclidean distances in the embedding space match the patristic distances on the backbone tree (see Jiang *et al.* 2022b for an extension to hyperbolic spaces).

While DEPP was successfully run on backbone trees with around 10 000 species, it has a fundamental scalability issue. DEPP requires calculating an $O(n^{2})$ distance matrix for training on a backbone tree with *n* leaves. When $n\gg 10^{4}$, saving the matrix (not to mention calculating it) is impossible on most machines. The alternative, to compute distances for each small batch during training, is too time-consuming and makes the training process too slow. Thus, DEPP is limited to training with roughly 10 000 species, making it unable to take advantage of the modern ultra-large reference sets.

This limitation is not just theoretical. In a recent effort, McDonald *et al.* (2023) built a new version of the widely used Greengenes (DeSantis *et al.* 2006) reference dataset (GG2) complete with a tree with 331 270 tips, including both genomes and 16S sequences. Because this tree is partially a species tree, placing 16S rRNA amplicon sequences on the tree is best done using DEPP. However, DEPP cannot train on such a large dataset. To use GG2, we needed to develop techniques that enable DEPP to scale to much larger backbone trees. These methods use divide-and-conquer to make the training time and memory grow quasi-linearly with the size of the backbone. However, as we show, much care is needed to retain the accuracy of the original method, mainly because of the trade-off between generalizability and precision during model training. This paper outlines various ways of scaling DEPP, culminating in a method called clustered-DEPP (C-DEPP), an earlier version of which is used in GG2. Besides motivating the C-DEPP design, our results shed light on more fundamental questions about applying phylogenetic-aware divide-and-conquer in the machine-learning context.

## 2 Methods

### 2.1 Preliminaries

Phylogenetic placement seeks to determine the optimal position of a query species on a backbone tree *T* consisting of *n* species accompanied by corresponding sequences. Note that the backbone tree is often inferred using longer sequences than what is used during placement (e.g., see Darling *et al.* 2014, Mahé *et al.* 2017, Upham *et al.* 2019). We also study the related tree-update problem: Given the backbone tree and the corresponding sequences, extend the tree to include the new species. Unlike placement, tree update produces a fully resolved tree that elucidates the relationships between queries. Jiang *et al.* (2022a) introduced the concept of *discordance* phylogenetic placement where the backbone tree is not solely or exclusively inferred from the sequences used for placement. For downstream applications, placing on the species tree is the ultimate goal, even when data from a single gene is available. In this paper, we focus on discordance placement and update and require that the backbone and query sequences are aligned.

We build on the metric learning method DEPP. Unlike traditional methods, which rely on predefined evolutionary models, it learns to embed sequences in Euclidean or Hyperbolic spaces such that the pairwise distances of the embeddings approximate the tree distances (Jiang *et al.* 2022b). In the training phase, DEPP uses stochastic gradient descent to set the parameters of the model to minimize the cost function:

(1) $$\underset{\Phi }{\text{arg max}}\sum_{i,j}\frac{1}{d_{ij}}{\left(||\Phi (s_{i})-\Phi (s_{j})|{|}_{2}-\sqrt{d_{ij}}\right)}^{2}$$

where *d_ij_* are the backbone tree distances and $\Phi (s_{i})$ are the output embeddings generated by the DEPP model. The model is an NN that consists of a single convolutional layer followed by a residual block, which comprises two convolutional layers with the input being added to the output. A final fully connected layer is appended to generate the embeddings. Once the model is trained on the backbone tree and MSA, we use it to place queries. For each aligned query sequence, we first use the model to embed the query. Then, we compute a distance vector between the query embedding and the backbone embeddings and use this distance vector as the input to the distance-based phylogenetic placement method APPLES-2 (Balaban *et al.* 2022), which finds the optimal placement.

Our goal is to extend DEPP to allow training with $n\gg 10^{4}$. Before introducing our empirically motivated method, we detail the datasets used throughout the paper for evaluation.

### 2.2 Training/testing datasets

We will focus on two datasets for benchmarking.

#### 2.2.1 Biological Web-of-Life dataset

The Web-of-Life (WoL) dataset, built by Zhu *et al.* (2019), contains 10 575 species and 381 marker genes. An ASTRAL (Mirarab *et al.* 2014) tree constructed using the 381 marker genes is available with the branch length calculated using sites sampled from the marker genes. Here, we use the 10 genes examined by Jiang *et al.* (2022b) as well as the marker 16S gene. Fragmentary sequences were removed for each gene by the original study. Because the dataset is at the limit of what DEPP can analyze, using this dataset, we can compare the effect of various scaling strategies to the baseline method trained on the entire dataset. To allow fair comparisons, we use the same set of queries used by Jiang *et al.* (2022b) for phylogenetic placement and tree update. For placement, 5% of the species of each gene are randomly selected as the queries and removed from the backbone. For query sequences, we use the same alignment to the backbone sequences as the one used to infer the reference tree; thus, our experiments do not directly test misalignment as a cause of placement error. For the tree update, we have two replicates. In each one, 100 random clades in the species tree ranging in size from 5 to 10 species were selected and pruned from the tree, with the remaining species serving as the reference. In total, across 11 genes, we have 5038 queries for placement and 12 248 queries for tree update.

#### 2.2.2 Simulated data

Balaban *et al.* (2023) generated a dataset comprising a species tree with 64 000 species and 100 genes undergoing extensive horizontal gene transfer (HGT) and some incomplete lineage sorting using Simphy (Mallo *et al.* 2016). Sequences were evolved with no insertion or deletion, omitting the need for aligning either references or queries. The rate of HGT is set such that the average discordance between gene trees and species is 0.38 (nRF). The branch lengths of the species tree were estimated under the GTR model using sequences from the 100 genes, with each gene providing 100 randomly selected sites. Here, we used only the first five genes for the placement experiments. The lengths of gene sequences vary between 375 and 616 bp. For identical sequences, a random species was retained and all others were removed. This step resulted in the removal of 505 to 1935 sequences among the five genes. Then, 5% of the species were randomly removed from the species tree as queries, resulting in 15 694 queries across all the genes.

### 2.3 Scaling DEPP using divide-and-conquer

**Figure 1.**
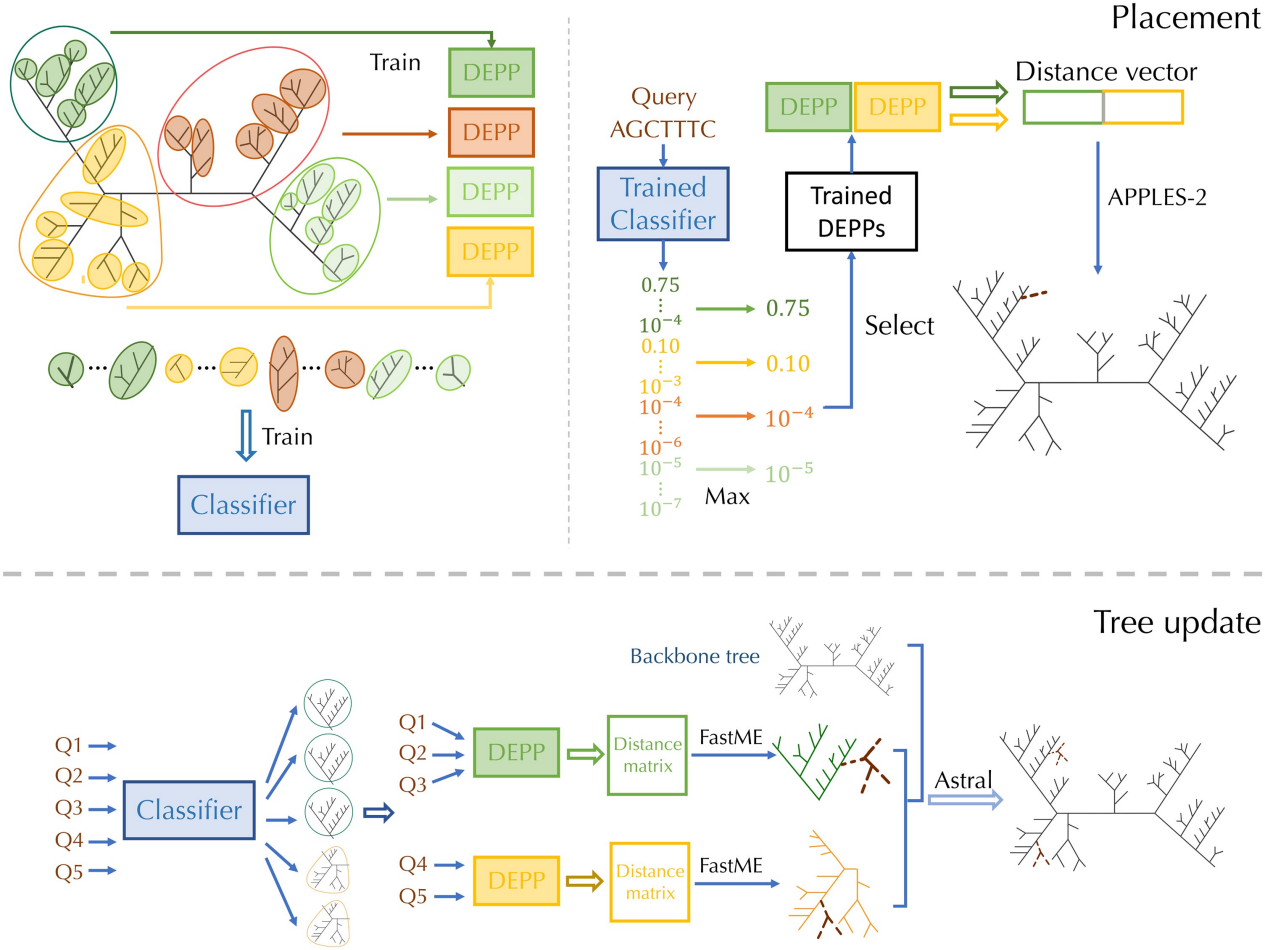
Pipeline of C-DEPP for phylogenetic placement and tree update. The tree is divided into multiple first-level groups and a separate NN is trained on each subset. Note that representatives from one group are added to the other ones during training (not shown here). Each first-level subset is further divided into smaller subsets. A classifier is trained to classify a query to one of these smaller subsets probabilistically. At the query time, probabilities from small subsets are summarized (max) to first-level subsets, and the corresponding models are used to calculate distances. APPLES-II is used to place on the backbone, using only distances from chosen models. Updates happen similarly, but using FastME to update subsets and using ASTRAL (as a supertree method) to combine the subset trees and the original backbone

Our proposed method C-DEPP uses a divide-and-conquer strategy to scale DEPP training. To summarize (Fig. 1), C-DEPP trains a separate model for each of several overlapping subtrees; for each query, C-DEPP uses a two-level classifier to select one or more subtrees, computes distances using those subtrees, and uses these distances as input to APPLES-II, leaving the other distances as missing. For this strategy to be accurate, many algorithmic tricks are needed. To motivate our final approach, we propose successively more advanced strategies and discuss the shortcomings of each. Since it is unclear how to evaluate these strategies theoretically, we resort to *empirical* evaluation (using the two datasets mentioned earlier) to show that each additional strategy contributes to better accuracy. The WoL dataset allows us to compare to normal DEPP while the simulated dataset allows examining the effects of having a very large training set and ground truth.

#### 2.3.1 Subsampling and random partitioning

**Figure 2.**
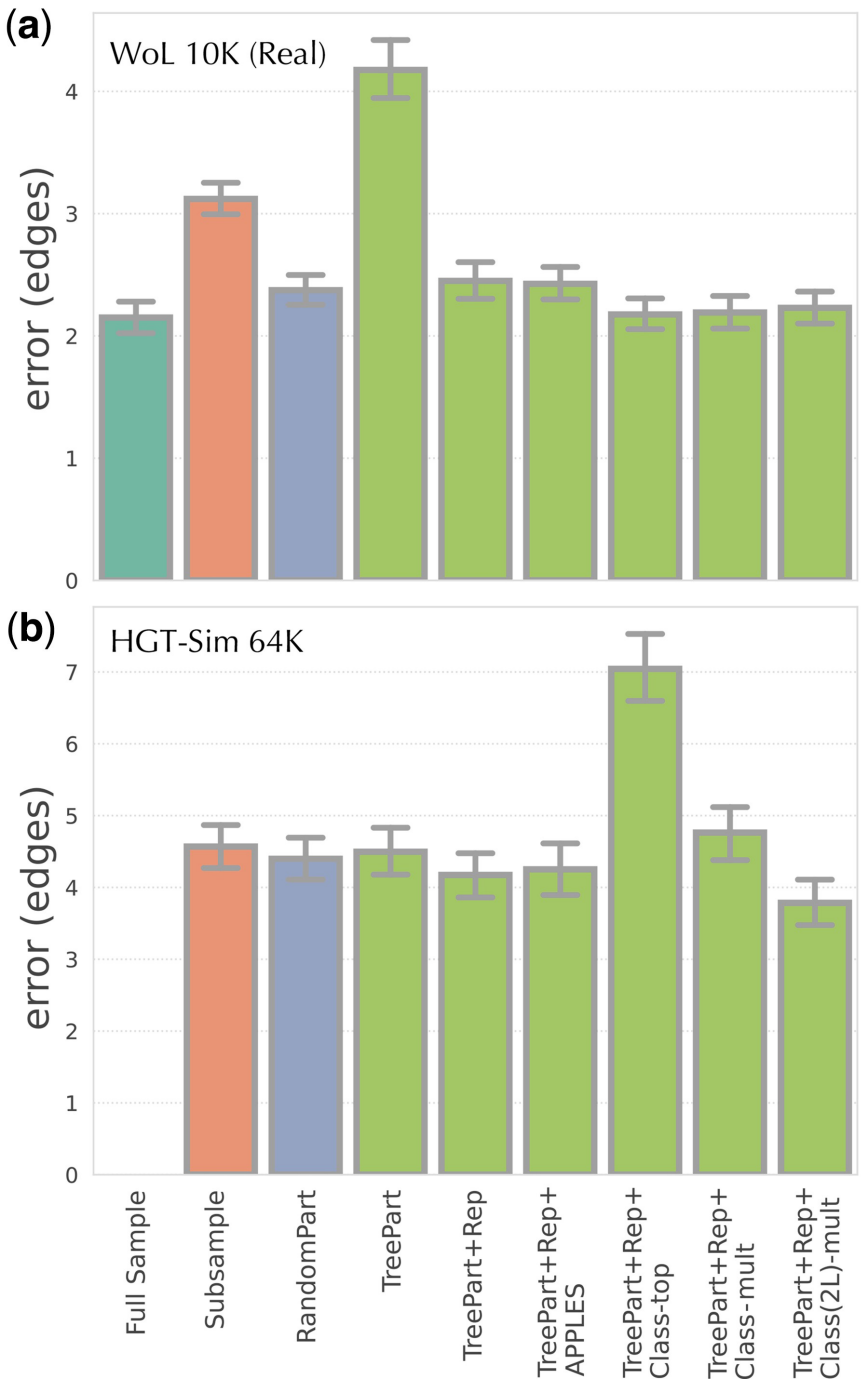
Comparing strategies for scaling DEPP, tested on (a) WoL dataset, averaged over the 11 marker genes and (b) an example gene of the simulated dataset with *n *=* *64 000 leaves. We show the mean and standard error of the placement error. The full dataset can only be used for training on the WoL dataset. We compare it with strategies of subsampling the dataset to 2500 (a) and 3500 (b) sequences (similar to partition sizes used in the remaining methods), partitioning the dataset randomly into 3 (a) and 17 (b) subsets, and C-DEPP. For C-DEPP, features are added sequentially; TreePart: tree-based clustering; Rep: augment clusters with representatives from other groups; Class: use a classifier to select one (-top) or multiple (-mult) clusters; Class(2L): two-level classification scheme. TreePart+Rep+Class(2L)-mult is the final version used elsewhere

The most obvious option for scaling is to simply train the model on a subset of species available in the tree. Such subsampling would still allow placement on the full tree as the model can embed the unused backbones as well. However, the accuracy of deep learning models is known to depend on the size of training sets. Moreover, taxon sampling is crucial to phylogenetic accuracy (Zwickl and Hillis 2002) and phylogenetic placement (Balaban *et al.* 2022). Thus, we expect subsampling to reduce the accuracy. On the biological WoL dataset, reducing sampling by tenfold increases the average error by around 50% (Fig. 2).

An alternative is to partition the data and train a separate encoder on each subset. The resulting *ensemble* model allows calculating distances to each backbone using the associated model. Implementing this ensemble model using a random partitioning of data is far better than subsampling (compare Subsample and RandomPart in Fig. 2) and comes close to the accuracy of the original model built on the full dataset. Nevertheless, it is less accurate and leads to the question: Does a more biologically motivated partitioning help accuracy?

#### 2.3.2 Tree-based partitioning

Instead of random partitioning, we can use the reference tree to create subtrees that are more evolutionary homogeneous. Such an approach creates a mixture of local experts, a well-established concept in machine learning (Jacobs *et al.* 1991), and used previously for phylogenetic placement (Mirarab *et al.* 2012). While many tree decomposition methods are available (e.g., Liu *et al.* 2011), we base our approach on the following criterion: divide the tree into subsets with <m leaves while minimizing the number of clusters. The optimal solution can be found in time linear in the size of the tree *n*, as implemented by the TreeCluster (Balaban *et al.* 2019) method. Thus, the tree is divided into subtrees $T=\{t_{1}\ldots t_{c}\}$, each with at most *m* species. We fix *m* (1500 by default) as *n* changes; thus, running time and memory both scale linearly with *n*.

Surprisingly, phylogenetic partitioning has a higher error than random partitioning (compare RandomPart and TreePart in Fig. 2). This reduced accuracy, which contrasts the rich history of divide-and-conquer phylogenetic methods, is likely related to specifics of deep learning: each model is trained on a subtree without the ability to learn from the full range of possibilities in the sequence space. Thus, the sequence embedder trained on the less diverse data is perhaps more precise but less generalizable (i.e., is overfit).

#### 2.3.3 Adding representatives (overlapping clusters)

To address the lack of generalizability, we design an approach that still uses the tree but creates overlapping training subsets: Each training subset includes *all* of the sequences in one of the phylogenetic partitions created previously plus a selection of sequences from other subsets. More precisely, we create another set of subtrees $\hat{T}=\{{\hat{t}}_{1}\ldots {\hat{t}}_{c}\}$ by adding *k* auxiliary species to each subtree in *T*. We set $k=\frac{1}{3}m\;\text{log}(c)$ by default to obtain sub-quadratic running time. Since we fix *m* when *n* grows, the size of the ${\hat{t}}_{i}$ subtrees grows with O(log(n)) and the total running time and memory grow with $O(n{\;\text{log}\;}^{2}(n))$. The auxiliary species added to *t_i_* are those with the minimum distances to *any* species in *t_i_*. We chose to add close species because APPLES2 uses only small distances when placing a query. By adding the auxiliary species close to a subset, we aim to help the placement of queries close to the boundary between clusters, which may have their closest relative outside the boundary. Since the subtrees are overlapping, the distance of a query to a reference species can be calculated using multiple models; when this happens, we take the median distance.

This overlapping partitioning approach performs far better than tree-based partitioning alone (compare TreePart+Rep to TreePart in Fig. 2). A reasonable explanation is that with auxiliary data added, while each model in the ensemble is still a local expert for a subtree, it is also aware of the larger context (e.g., sequences outside the subtree). Thus, its embeddings are better than other models for sequences belonging to that subset but it also is not ignorant of the rest of the space. Such hybrid approaches have been used previously in machine learning (Peralta *et al.* 2019, Liao *et al.* 2019).

#### 2.3.4 Selecting the best model(s): classifiers

When analyzing a query, instead of simply using all distances computed from all the models, would it be better to pick the best “expert” model and use only its distances? Note that APPLES-II allows missing distances, enabling us to place on the full tree using distances computed from a single model. Similar to methods such as SCAMPP and pplacer-XR (Wedell *et al.* 2021, 2023), we can use an initial placement using APPLES+JC to pick a subset and use only the model trained on that subset for placement. Empirically, this approach works no better or worse than giving APPLES-II the distances from all the models (compare TreePart+Rep+APPLES and TreePart+Rep in Fig. 2). But can we do better than using an initial placement?

Deciding which cluster to use can itself be posed as a classification problem where the input is a sequence, and the output is a probability vector indicating the likelihood of the input sequence belonging to each subtree. Using an architecture very similar to DEPP, we designed such a classifier. The only difference compared to DEPP is that the number of embedding dimensions equals the number of partitions and the final output goes through a softmax layer that ensures the *L*_1_ norm of the output is 1 (i.e., can be interpreted as the probability of the partition). The loss function is the cross-entropy between the output probabilities and the ground truth (i.e., indicator function of the correct partition).

Using this classifier and simply picking the most likely cluster improves accuracy in the real dataset but dramatically reduces the accuracy in the simulated high-HGT dataset (TreePart+Rep+Class-Top versus TreePart+Rep in Fig. 2). We next resort to using multiple models when they all have a substantial likelihood. More precisely, we sort the models based on their likelihood and take each of the top four models if it has a likelihood at least 1/200 times the likelihood of the previously taken model (the threshold was picked arbitrarily and not optimized). This strategy substantially improves results (TreePart+Rep+Class-Multi in Fig. 2) but still remains slightly worse than using all the models on the simulated dataset. We believe the reason is that the classifier needs to assign a sequence to very large groups, a task that may be difficult in the face of HGT among distantly related species. Recall that the backbone tree (used in partitioning) is the species tree and may not reflect the relationships among genes, making classification to large subtrees less accurate, including cases with high confidence for the wrong class. Using smaller classes could solve this issue but would be detrimental to training accurate embedded models.

#### 2.3.5 2-Level classifier

We propose a two-level classification scheme. For the second level, each subtree in *T* (e.g., *t_i_*) is further split into smaller subtrees ($T_{i}^{\prime }=\{t_{1}^{i}\ldots t_{c_{i}}^{i}\}$) with a maximum of m′ leaves, where m′ is set to 30 by default. Second-level (smaller) subsets are used for classification while the larger first-level subsets are used for creating embedders. Thus, the classifier is trained to select among the second-level subtrees $\underset{i}{\cup }T_{i}^{\prime }$. This classifier is built identically to what we described earlier for one-level classifiers. To determine the first-level subtree of a query (i.e., the large class), first, the classifier is used to calculate the likelihood $p_{j}^{i}$ of the query belonging to each second-level subtree *j* of first-level subtree *i*. Next, we define the score of the first-level subtree *t_i_* to be sti=maxj pji and use these scores to assign the query to the first-level subtree(s) with the highest score(s). We keep assigning the query to up to four subtrees with the highest scores as long as the subset has a score that is at least 1/200 of the score of the previous subtree. Note that the second-level classifiers are only used to select the first-level classes. Once the query is assigned to the first-level subtree(s), it is placed using distances calculated using the corresponding first-level embedder models. The role of the second-level classifiers is to better reveal classification uncertainty, which can be underestimated when we train classifiers directly on large classes.

The 2-level classification strategy retains the same accuracy as the 1-level for the real dataset and substantially improves the accuracy for the simulated dataset (Fig. 2). This version of the tool including all these features is the final version we will use throughout the rest of the paper as C-DEPP.

### 2.4 Experimental details

To evaluate the performance of C-DEPP, we compare it to leading alternatives for both placement and update tasks.

For placement, we measure the number of edges between the placement and the correct placement on the tree. For the simulated dataset, we use the true species tree as the backbone, and thus, the correct placement is well-defined. On the real dataset, we take the position of the species in the original ASTRAL tree inferred from all 381 marker genes (before removing the queries) as the optimal position. We compare DEPP (only tested on the smaller WoL dataset) and C-DEPP to the following methods.

EPA-ng (Barbera *et al.* 2019) performs maximum-likelihood phylogenetic placement. We use RAxML-ng (Kozlov *et al.* 2019) to infer the parameters of the substitution models and the backbone tree under the GTR+Γ model.
EPA-ng-SCAMPP (Wedell *et al.* 2023) is a method that enables EPA-ng to work on ultra-large trees by first finding a subtree and placing on that subtree using EPA-ng. We apply the default backbone tree size of 2000. Similar to EPA-ng, we use RAxML-ng to prepare the backbone parameters under the GTR+Γ model. The placement with the highest support is selected for each query.
APPLES2+JC: We use APPLES-2 (Balaban *et al.* 2022) which uses Jukes–Cantor (JC) model to estimate distances. We use RAxML-ng under JC models to recalculate the branch length of the input backbone tree for this tool.
RAPPAS (Linard *et al.* 2019) is a *k*-mer based method. We employ RAxML-ng to reestimate the branch length of the species tree and build the database. The branch lengths are computed from the corresponding single-gene MSA under GTR+Γ model.

For tree updates, we measure both the Robinson and Foulds (1981) (RF) and quartet distance between the true/reference tree and the inferred updated tree. We compare these methods.

RAxML (Stamatakis 2014) maximum-likelihood inference is used to update an existing tree; we use the backbone tree as a constraint to fix its topology. When multiple genes are available, we concatenate the sequences from all the genes.
JC+FastME. We first calculate the distances between all pairs of the sequences under the JC model and use the distance matrix as the input to the distance-based FastME (Lefort *et al.* 2015). When using more than one gene, we first calculate the distance matrix for each gene and then take the median of the distances for each pair across all genes to summarize the distance matrices. Distances among pairs of backbone species are fixed to patristic distances in the backbone tree to encourage FastME to keep the backbone relationships fixed.
DEPP+FastME is run using a pipeline similar to JC+FastME, using DEPP models to estimate the distances between sequences rather than the JC model.
C-DEPP+FastME first trains the models from the backbone and then performs three steps (Fig. 1). First, we assign each query to the first-level subtree with the highest score *s_i_*; when multiple genes are available, we simply average the scores across genes. We calculate the distance matrices for all query and backbone species in each subtree using all genes. As in the previous methods, we take the median across genes and fix distances among backbone species to their patristic distance on the backbone tree. Whether we have single or multiple genes, we obtain a single distance matrix at the end for each subtree. We then re-infer each subtree using FastME given this distance matrix. To combine all the subtrees into a full tree, we give updated subtrees as well as the backbone tree as input to ASTER software (Zhang and Mirarab 2022) without weighting. Note that, here, we use ASTER as a supertree method and not as a summary method combining gene trees.

All methods require an MSA for references, and all methods except for RAPPAS require aligning queries to references. On the WoL dataset, we use the existing reference MSA, and sites with over 95% gaps are removed. Queries are not re-aligned after removal from the reference. For simulated data, sequences contain no indels and thus are already aligned. Therefore, our study does not directly test the impacts of alignment error, which may disadvantage RAPPAS, the only method that does not require an alignment of queries.

## 3 Results

### 3.1 Simulated data

**Figure 3.**
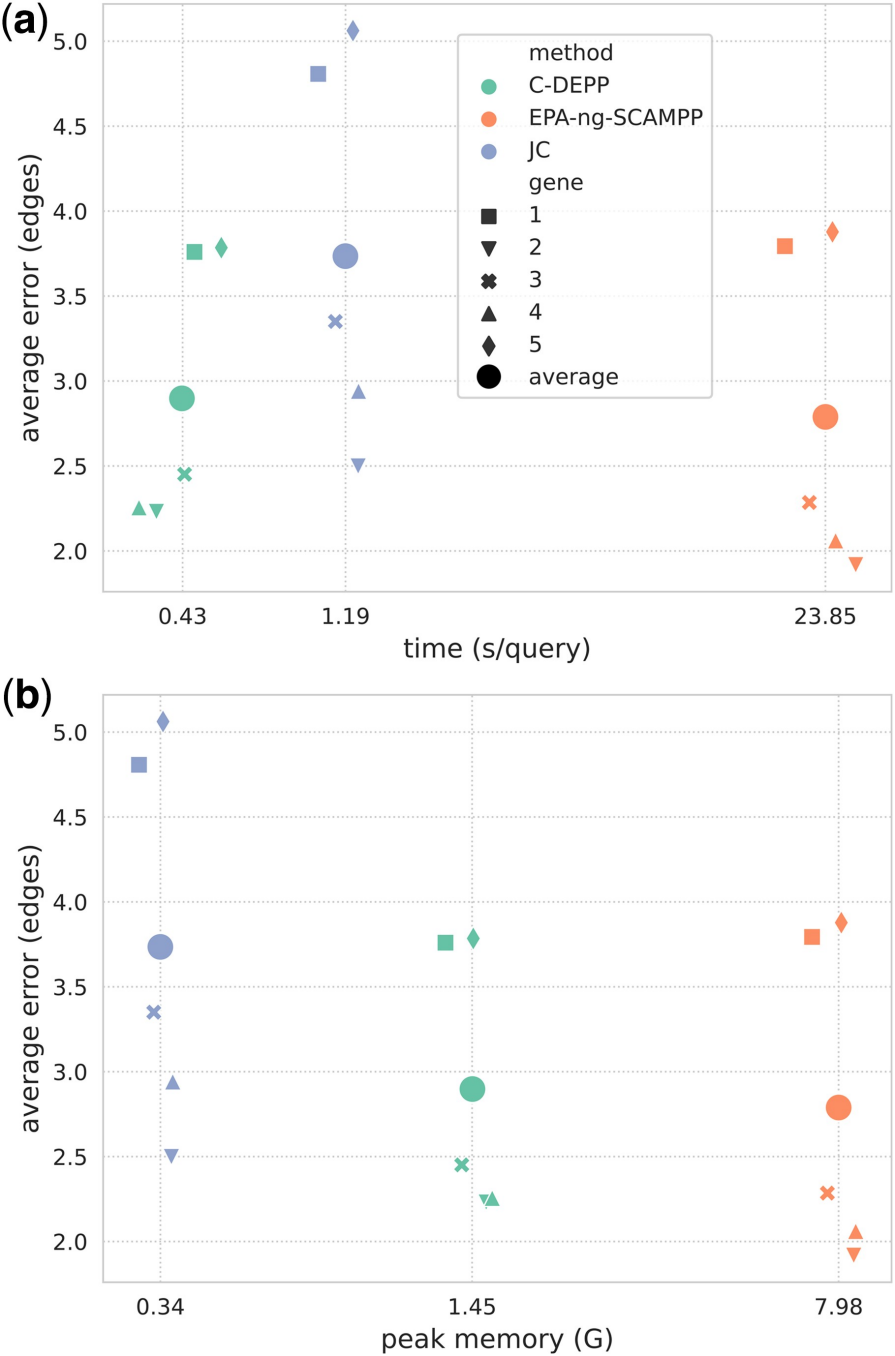
Results on HGT-Sim (64K) comparing average placement errors (*y*-axis) with running time (a) and peak memory (b). Larger dots are averages over all five genes. The *x*-axis is in the log-scale

We start by evaluating C-DEPP on simulated data. Note that on this dataset, DEPP would require more than 250 GB of memory for training, and hence, we could not run it. Among other methods, EPA-ng-SCAMPP has the best placement accuracy closely followed by C-DEPP (Fig. 3). Specifically, EPA-ng-SCAMPP has an average error of 2.78 edges over all five genes (with an average of 59 576 backbone leaves) compared to 2.89 edges for C-DEPP. Both of the two methods are substantially more accurate than APPLES+JC with an average error of 3.73 edges. When evaluating the entire distribution of the error, the trend is generally consistent but a long tail of high errors is observed for all methods (Fig. S1). EPA-ng-SCAMPP finds placements at most three edges away from the correct placement 91.7% of the time, which is 2.4% higher than C-DEPP and 11% higher than APPLES+JC.

**Figure 4.**
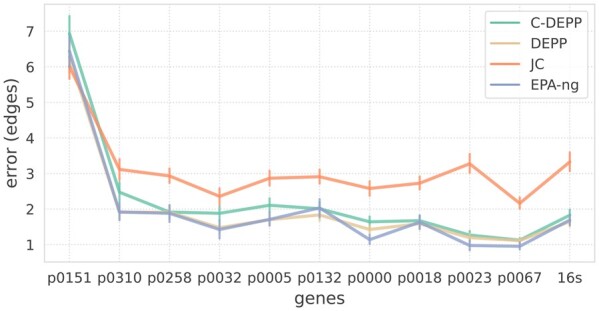
Placement error, showing mean and standard error of error, across 11 genes of the WoL dataset with $\approx 10^{4}$ species, where DEPP can be run

While slightly more accurate, EPA-ng-SCAMPP has much higher computational demands in terms of both running time and memory. Both C-DEPP and APPLES+JC are much faster than SCAMPP-EPA-ng (Fig. 3). Specifically, C-DEPP is 50 times faster than EPA-ng-SCAMPP, with a slightly higher error of 0.1 edges. Interestingly, C-DEPP is also faster than the less accurate method APPLES+JC. In terms of memory consumption, APPLES+JC is the most efficient requiring less than 0.5 GB, followed by C-DEPP which requires less than 1.5 GB; EPA-ng-SCAMPP required 8 GB on this dataset.

Compared to the *k*-mer-based method RAPPAS, C-DEPP demonstrates significantly higher accuracy. C-DEPP exhibits an average error of 3.8 edges, whereas RAPPAS yields an average error of 17.9 (Fig. S3). Furthermore, RAPPAS displays a greater number of outliers. Specifically, out of 3110 queries, RAPPAS produces 1241 placements with errors exceeding 30, whereas C-DEPP only has 204 such instances. The low accuracy of RAPPAS may be due to its strict molecular clock assumptions and sensitivity to high branch length heterogeneity (which our tree shows). In terms of the efficiency of placement, C-DEPP outperforms RAPPAS by a considerable margin. In the testing stage, C-DEPP completes its analysis in 8 min, while RAPPAS requires 108 min. Additionally, placement using C-DEPP is substantially more memory-efficient, utilizing only 1.9 GB compared to RAPPAS’s 7.9 GB. In this comparison, only the first gene was considered due to RAPPAS’s inability to construct the database for the other four genes given 250 G of memory and a wall time limit of 48 h.

### 3.2 Biological data

#### 3.2.1 Placement

Averaged over all 11 genes, the performance of EPA-ng and DEPP is comparable with 2.0 edges of error on average (Fig. 4). Compared to DEPP, C-DEPP has only a slightly higher average error (2.2 edges). DEPP and C-DEPP are close in accuracy for most genes, but DEPP has a noticeable advantage for four genes; for these genes, accuracy was similar across most queries, except for a handful of outlier queries with high levels of missing data (gaps in the sequences) and high placement errors (Fig. S2). All three methods are significantly more accurate than APPLES+JC (3.1 edges of error).

While maintaining the high accuracy of DEPP almost intact, C-DEPP significantly reduces training time. For instance, training the DEPP model on 16S data from the WoL dataset using a Tesla V100-SXM2-32GB card takes 210 min. In contrast, the C-DEPP model requires only one-third of that time to train on the same data. In terms of testing, while C-DEPP has a longer running time than DEPP, it is more memory-efficient. For example, when placing 1000 16S sequences onto the backbone tree with 7400 leaves, C-DEPP has a peak memory usage of 0.67G (126 s running time), while DEPP requires a peak memory usage of 1.5G (26 s), reducing memory consumption by more than half compared to DEPP’s. On much larger datasets, the memory requirements of DEPP (which grow quadratically) would become prohibitive.

#### 3.2.2 Tree update

**Figure 5.**
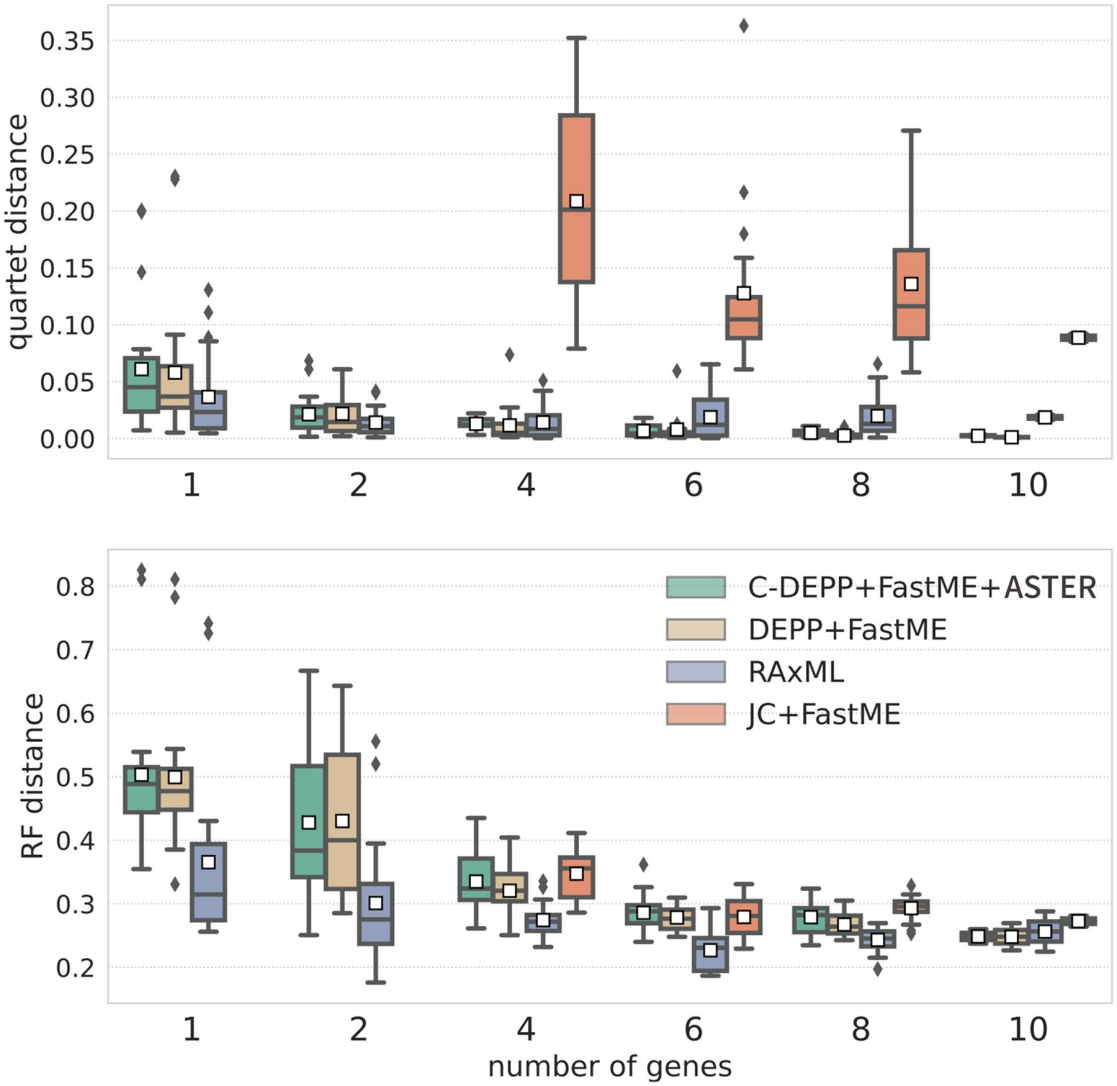
Tree update results on the WoL dataset. We show both quartet and RF distances, only restricted to query taxa, as the number of genes increases. FastME fails to run in some cases with one or two genes because specific pairs of sequences occasionally have no overlapping nongap sites

When using a single gene to update a tree, RAxML has a clear advantage over other methods with lower error measured by quartet distance or RF distance (Fig. 5). However, C-DEPP has very similar accuracy to DEPP. When multiple genes are used, patterns gradually change. Errors drop rapidly for DEPP and C-DEPP but not the concatenation-based RAxML. For example, when using two genes, the average quartet distance of C-DEPP is 0.02 which is around one-third of its error with one gene. The quartet error quickly drops down to 0.006 with six genes. The pattern is similar (though less pronounced) when examining the RF distances. For example, the RF distances are reduced by half from using a single gene to using six genes. In contrast, the error reduction is less pronounced for RAxML; the quartet error does not reduce notably in response to increasing the number of genes beyond two and can occasionally increase C-DEPP and DEPP start to outperform RAxML with six genes or more when measuring the quartet distance or with 10 genes when measuring the RF distance. The performance of JC+FastME is significantly worse than the other methods measured by quartet distance and is the worst or among the worst methods measured by RF distance. Finally, note that DEPP and C-DEPP have very similar accuracy. When the dataset is small enough to allow running both, there is no benefit in using C-DEPP on this dataset. However, for larger datasets, C-DEPP is the only option possible due to its size.

### 3.3 Application: C-DEPP used on GG2

Recently, McDonald *et al.* (2023) inferred a reference tree combining ≈16 000 genomes and 321 210 16S full-length sequences to produce the second version of the popular Greengenes database. The next goal of that project was to place all 23 113 447 short V4 16S rRNA Deblur v1.1.0 8 (Amir *et al.* 2017) amplicon sequence variants from Qiita (Gonzalez *et al.* 2018) (retrieved 14 December 2021) on this tree. Since the backbone tree is a mixed species/gene tree, DEPP, which can learn to place on any tree, was the appropriate placement method. However, because the backbone tree was more than an order of magnitude larger than what DEPP could handle, we had to develop the C-DEPP approach studied here (an earlier version akin to TreePart+Class-Multi).

As the query sequences are hybrid, encompassing full-length 16S, complete V4 region, and a segment of the V4 region, we developed four distinct models tailored to different scenarios: one for full-length 16S, another for the V4 region of 16S (240 bp), and two more for V4 segments, with lengths of 150 and 100 bp. Multiple models are trained for different regions as evidence shows that the performance of DEPP would degrade if used with highly gappy aligned queries (Jiang *et al.* 2022a). For each query sequence, we first align it to the backbone MSA using UPP (Nguyen *et al.* 2015). We aligned full-length 16S queries with the backbone full-length 16S MSA and aligned all short-read 16S sequences with the backbone MSA of the full V4 region. If the queries contain regions not present in the reference sequences, those specific regions are ideally marked as insertion sites by UPP, which are omitted from the alignment before we proceed. For 16S reads, we selected the appropriate model (240, 150, or 100 bp) based on the query alignment. We chose the model corresponding to the longest region, as long as the proportion of gaps in its alignment was below 0.2. Queries with a gap proportion exceeding 0.2 in all three cases were filtered from final analyses, removing 10% of the queries.

**Figure 6.**
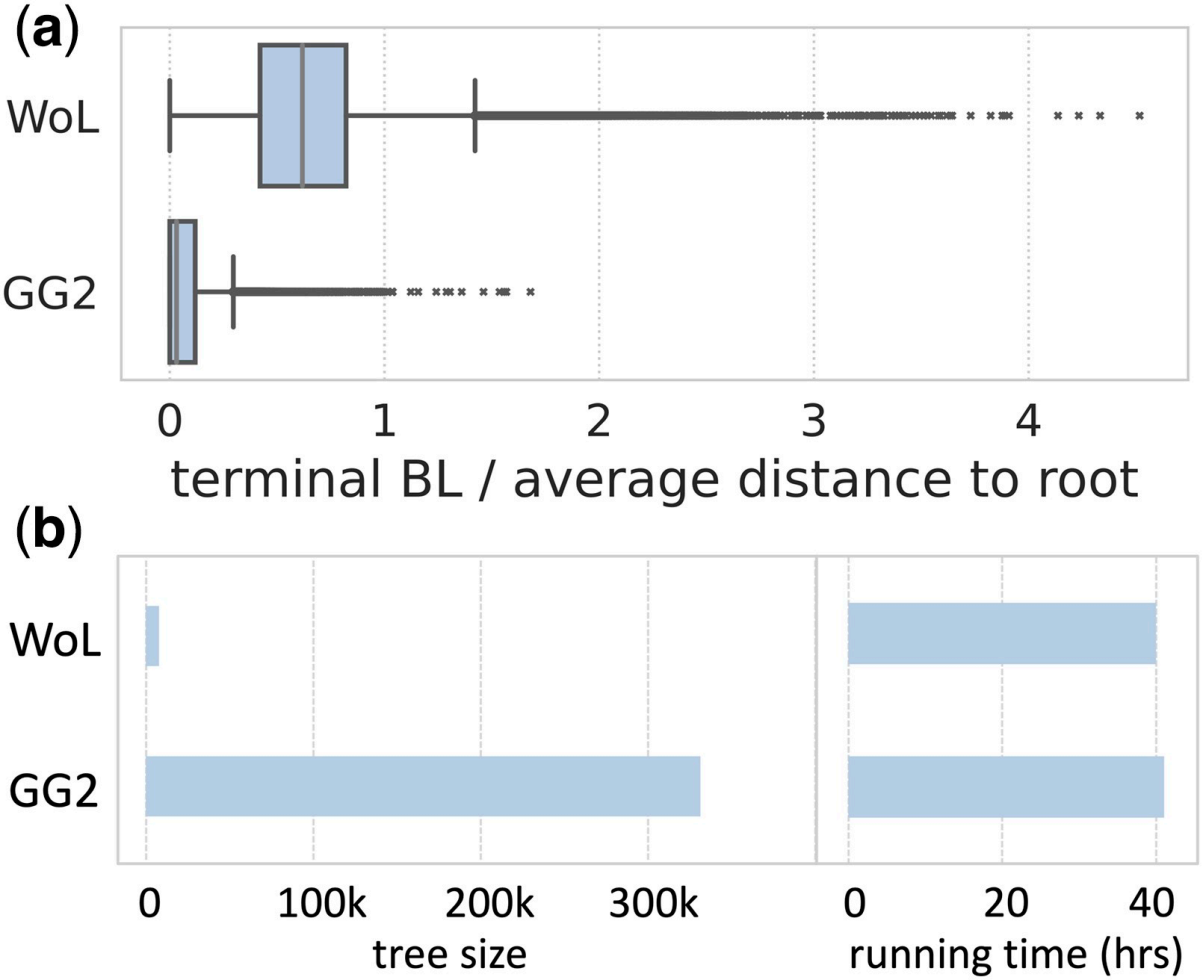
Impact of using C-DEPP on GG2 reference set with the THDMI dataset. (a) Terminal branch length of the leaves in WoL reference tree and GG2 reference tree. (b) Statistics of placement onto WoL reference tree and GG2 reference tree. Running times include alignment using UPP on a machine with 128 cores.

McDonald *et al.* (2023) extensively report on the results of that analysis showing improved taxonomic classification and consistency across data types in using GG2. Here, instead of repeating those results, we focus on the impact of C-DEPP allowing a backbone tree of size > 3 × 10^5^. Using the original backbone of size 10^4^ leads to placements with much longer terminal lengths for the pendant edges (Fig. 6a). The reduced terminal branch lengths of C-DEPP confirm the motivation for using large backbone trees: they can provide more precise phylogenetic placements, which can then enable a better understanding of the microbiome compositions. Incredibly, using this much larger tree did not impact running time. Placement using C-DEPP onto the backbone tree with more than 330 000 leaves requires roughly the same time as placement onto the WoL tree (with 10 575 leaves) using DEPP, further demonstrating the scalability of C-DEPP (these reported running times include alignment using UPP).

## 4 Discussions

To enable the machine-learning placement method DEPP to scale to ultra-large datasets, we introduced C-DEPP. C-DEPP is not more accurate than DEPP but is far more scalable as the backbone size grows. The time and memory of C-DEPP grow quasi-linearly with the backbone size, improving on quadratic scaling of DEPP. As more microbial genomes become available, sub-quadratic methods become increasingly needed. The C-DEPP method has already been used (McDonald *et al.* 2023) and fills an important practical gap. Using APPLES-II with JC distances is scalable but not accurate enough for placing on species trees while maximum likelihood is too slow. C-DEPP is as salable as simple distance-based methods but comes close to maximum likelihood in terms of accuracy.

In designing C-DEPP, we used divide-and-conquer techniques with two key twists. First, we augmented phylogeny-based clusters by representatives from other groups. This is helpful because sequence embedders need to “see” sufficiently diverse examples to learn generalizable models. This benefit may be particular to machine learning and is very different from the use of overlapping subsets in supertree methods (e.g., Nelesen *et al.* 2012), which do so to enable the merging of subtrees. Second, we found it mildly beneficial to restrict distance calculation to models most likely to have generated a query *only* if a two-layer classifier is used. A biological insight underpins this two-layer design. The main cause of gene tree discordance on microbial datasets is HGT, which can hamper classifying a gene sequence into groups defined on the species tree. Using large classes can mask discordance and lead to high confidence for the wrong subtree. By using smaller subtrees, we enable the classifier to find clades in the species tree corresponding to the HGT donor and recipient in a specific gene, reducing cases of highly confident but incorrect classifications. We test this hypothesis by examining the correlation between error differences and the level of HGT. We quantify HGT by computing the average gene tree distances of the 10 nearest neighbors in the species tree. Results show that among the queries where 2-level classification is dramatically better, the gene trees are more discordant to the species tree (Fig. S4), indicating that those queries are more likely to be affected by HGT.

Another factor that impacted accuracy was the query novelty quantified using its distance to its closest species in the backbone tree. We observed a consistent performance decline across all methods as query novelty increases (Fig. S5). On simulated data, C-DEPP exhibits comparable performance with EPA-ng, even with queries reasonably distant from the backbone species (e.g., in the 0.2–0.8 range). According to an ANOVA test, there was no statistically significant difference between the two methods (*P *=* *.22) and the novelty level had no significant impact on the relative accuracy of the methods (*P *=* *.14). Similarly, the comparison to JC did not depend on the novelty level in a significant way (*P *=* *.87). In the WoL dataset, increasing the novelty level had no significant impact on the relative accuracy of C-DEPP and EPA-ng (*P *=* *.75) but it did impact the comparison to JC (*P *=* *.00004). Thus, the effectiveness of C-DEPP in comparison to other methods does not diminish with more novel queries.

Both DEPP and C-DEPP can improve in the future in several ways. For the tree update, alternative pipelines should be explored. For example, instead of summarizing across genes by computing the median distance, we can compute an updated tree per gene and let ASTRAL combine them. We also did not force the backbone trees to be fixed. Imposing such constraints on ASTRAL may improve the accuracy. Another limitation of the current (C-)DEPP methodology is that its accuracy can degrade as the portion of gaps in the query alignment increases; while Jiang *et al.* (2022a) proposed a model (not used in our experiments) to reconstruct gaps, creating models more robust to highly gappy queries can enable us to train one model on the full-length MSA and place reads aligned to that MSA. Another avenue for improvement is incorporating support. Recently, Hasan *et al.* (2022) and Rachtman *et al.* (2022) have shown various ways of computing support for distance-based placement using APPLES. However, C-DEPP has not incorporated uncertainty calculation. Subsampling techniques similar to Jiang *et al.* (2022a) can be incorporated into C-DEPP, but such measures of support need to account for classification errors. This can be achieved using the probability output from the classifier as an indicator of uncertainty.

Our study also leaves some questions unanswered. We did not explore the impact of alignment error, nor did we study the impact of gaps. Jiang *et al.* (2022a) have explored both for DEPP but the impact on the classification step of C-DEPP needs further study. Also, the most accurate method, SCAMPP, was slow in our analyses. A new version called Batch-SCAMPP (Wyyreferenceedell *et al.* 2022) promises much faster running times. In preliminary tests, we saw some improvements in speed but not enough to bridge the gap with C-DEPP. Batch-SCAMPP takes between 2.5 and 9 h on the 64k dataset, depending on the level of HGT in a gene, compared to 12 h for normal SCAMPP. In contrast, C-DEPP takes around 30 min. Finally, while here we scaled to trees with up to 330 000 leaves, larger trees are being constantly built. In our future applications of C-DEPP, we plan to use it to place queries on trees with millions of leaves.

## Supplementary Material

btae361_Supplementary_Data

## Data Availability

All data used for the analyses in this article are publicly avaliable at https://github.com/yueyujiang/dataset_cdepp/.
