# Supplementary material for: Scaling DEPP phylogenetic placement to ultra-large reference trees: a tree-aware ensemble approach
Source: Bioinformatics. 2024 Jun 13;40(6):btae361. doi: 10.1093/bioinformatics/btae361 (PMC11193062; doi:10.1093/bioinformatics/btae361)
Supplement: btae361_Supplementary_Data [file btae361_supplementary_data.pdf]

867

Supplementary material: C-DEPP

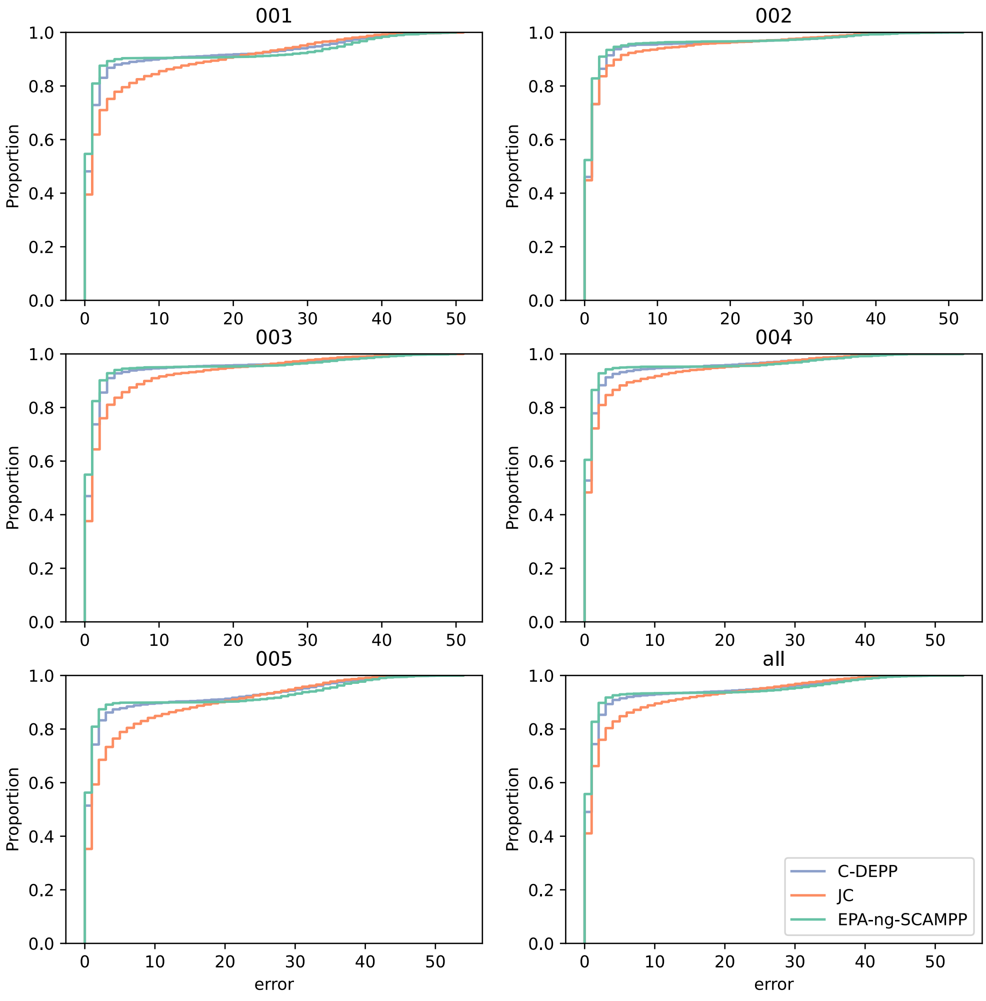

Fig. S1. Empirical cumulative distribution functions (ECDF) of errors in HGT-Sim (64K) dataset

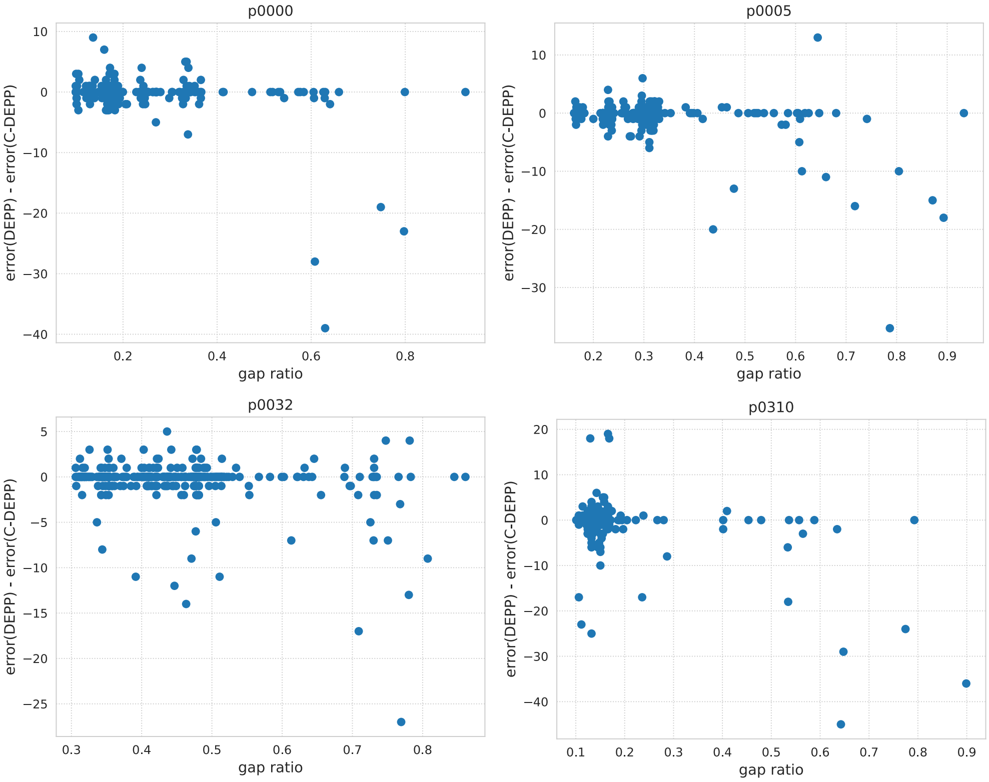

Fig. S2. Impact of gap ratio (proportion of query sequence that are gaps) to the difference of placement error between DEPP and C-DEPP on four maker genes (p0000, p0005, p0032, p0310) where DEPP substantially outperformed C-DEPP; see Figure 4 of the main paper. Note that the outlier queries with much higher placement errors tend to have very high levels of gappiness.

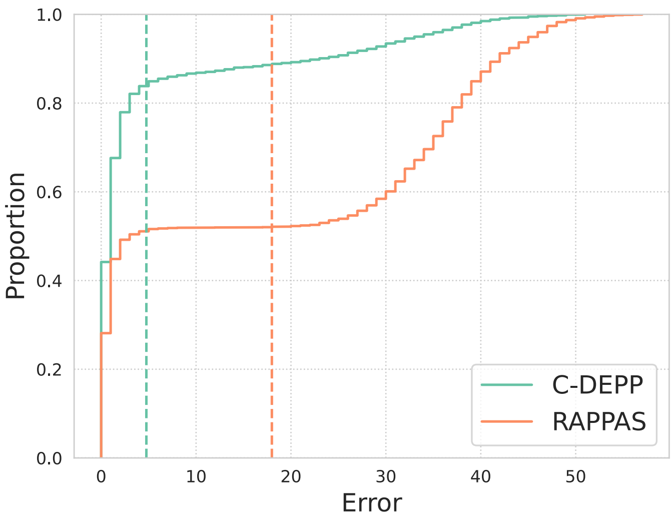

Fig. S3. Placement error of C-DEPP and RAPPAS on the first gene of the simulated data. The vertical lines are the average error over the 3110 queries.

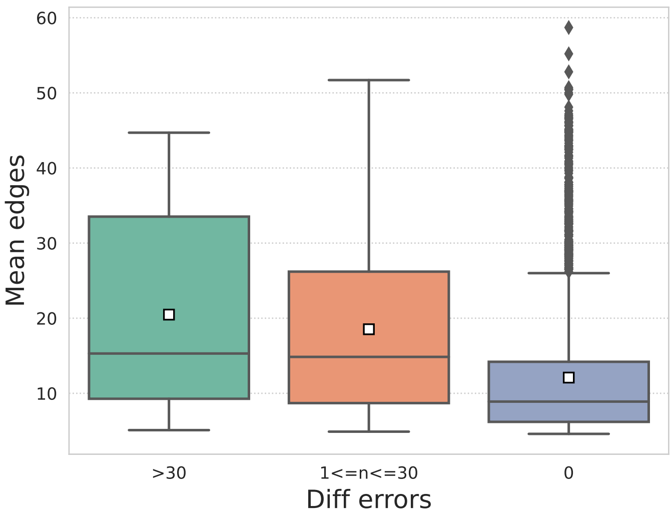

Fig. S4. Error differences using two-levels (2L) classification v.s. using one-level (1L) classification. y-axis: HGT measured by the average gene tree distances of the 10 nearest neighbors in the species tree. Both the gene tree and species tree distances are calculated by the number of edges. Left box: the data of queries with 1L errors higher than 2L errors for more than 30 edges (52 queries); middle box: data of queries with 1L errors higher than 2L errors for 1 to 30 edges (280 queries); right box: the data of queries with the same error using 1L or 2L (2368 queries). The results use data from the first gene of HGT simulated data (3110 queries in total).

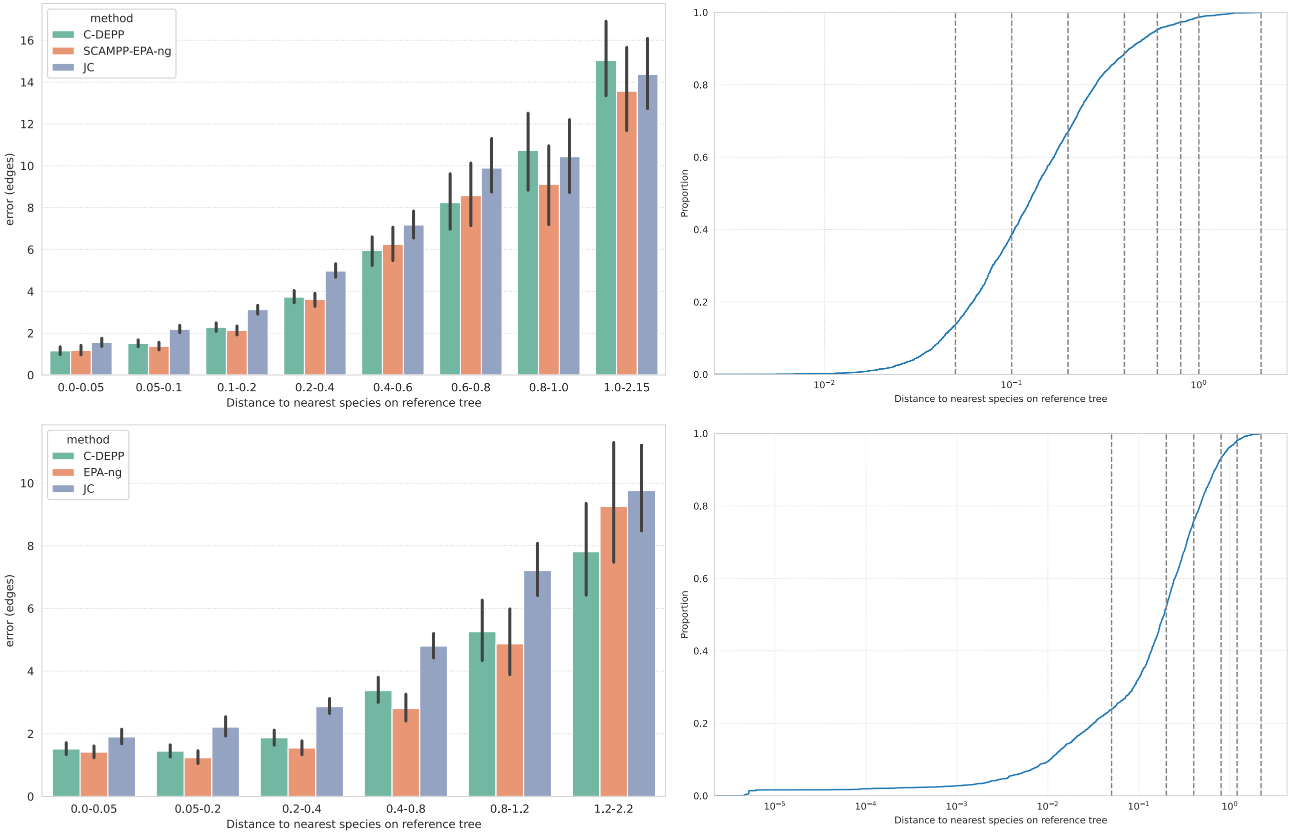

Fig. S5. Left: Impact of query novelty. Query novelty is assessed based on the distance between the query and its closest species in the backbone tree; Right: ECDF of query novelty. The vertical dash lines are the right boundaries of the intervals in the left figure. Top: results from the (64K) simulated dataset. Bottom: results from the Biological data.
